# Chemoproteomics reveals Toll-like receptor fatty acylation

**DOI:** 10.1186/s12915-014-0091-3

**Published:** 2014-11-05

**Authors:** Nicholas M Chesarino, Jocelyn C Hach, James L Chen, Balyn W Zaro, Murugesan VS Rajaram, Joanne Turner, Larry S Schlesinger, Matthew R Pratt, Howard C Hang, Jacob S Yount

**Affiliations:** Department of Microbial Infection and Immunity, Center for Microbial Interface Biology, The Ohio State University, Columbus, OH 43210 USA; Biomedical Informatics, Internal Medicine in the Division of Medical Oncology, The Ohio State University, Columbus, OH 43210 USA; Departments of Chemistry and Molecular and Computational Biology, University of Southern California, Los Angeles, CA 90089 USA; Laboratory of Chemical Biology and Microbial Pathogenesis, Rockefeller University, New York, NY 10065 USA

**Keywords:** Palmitoylation, Post-translational modification, Click chemistry, Toll-like receptor, TLR2, Fatty acylation, Proteomics

## Abstract

**Background:**

Palmitoylation is a 16-carbon lipid post-translational modification that increases protein hydrophobicity. This form of protein fatty acylation is emerging as a critical regulatory modification for multiple aspects of cellular interactions and signaling. Despite recent advances in the development of chemical tools for the rapid identification and visualization of palmitoylated proteins, the palmitoyl proteome has not been fully defined. Here we sought to identify and compare the palmitoylated proteins in murine fibroblasts and dendritic cells.

**Results:**

A total of 563 putative palmitoylation substrates were identified, more than 200 of which have not been previously suggested to be palmitoylated in past proteomic studies. Here we validate the palmitoylation of several new proteins including Toll-like receptors (TLRs) 2, 5 and 10, CD80, CD86, and NEDD4. Palmitoylation of TLR2, which was uniquely identified in dendritic cells, was mapped to a transmembrane domain-proximal cysteine. Inhibition of TLR2 S-palmitoylation pharmacologically or by cysteine mutagenesis led to decreased cell surface expression and a decreased inflammatory response to microbial ligands.

**Conclusions:**

This work identifies many fatty acylated proteins involved in fundamental cellular processes as well as cell type-specific functions, highlighting the value of examining the palmitoyl proteomes of multiple cell types. S-palmitoylation of TLR2 is a previously unknown immunoregulatory mechanism that represents an entirely novel avenue for modulation of TLR2 inflammatory activity.

**Electronic supplementary material:**

The online version of this article (doi:10.1186/s12915-014-0091-3) contains supplementary material, which is available to authorized users.

## Background

**Figure 1:**
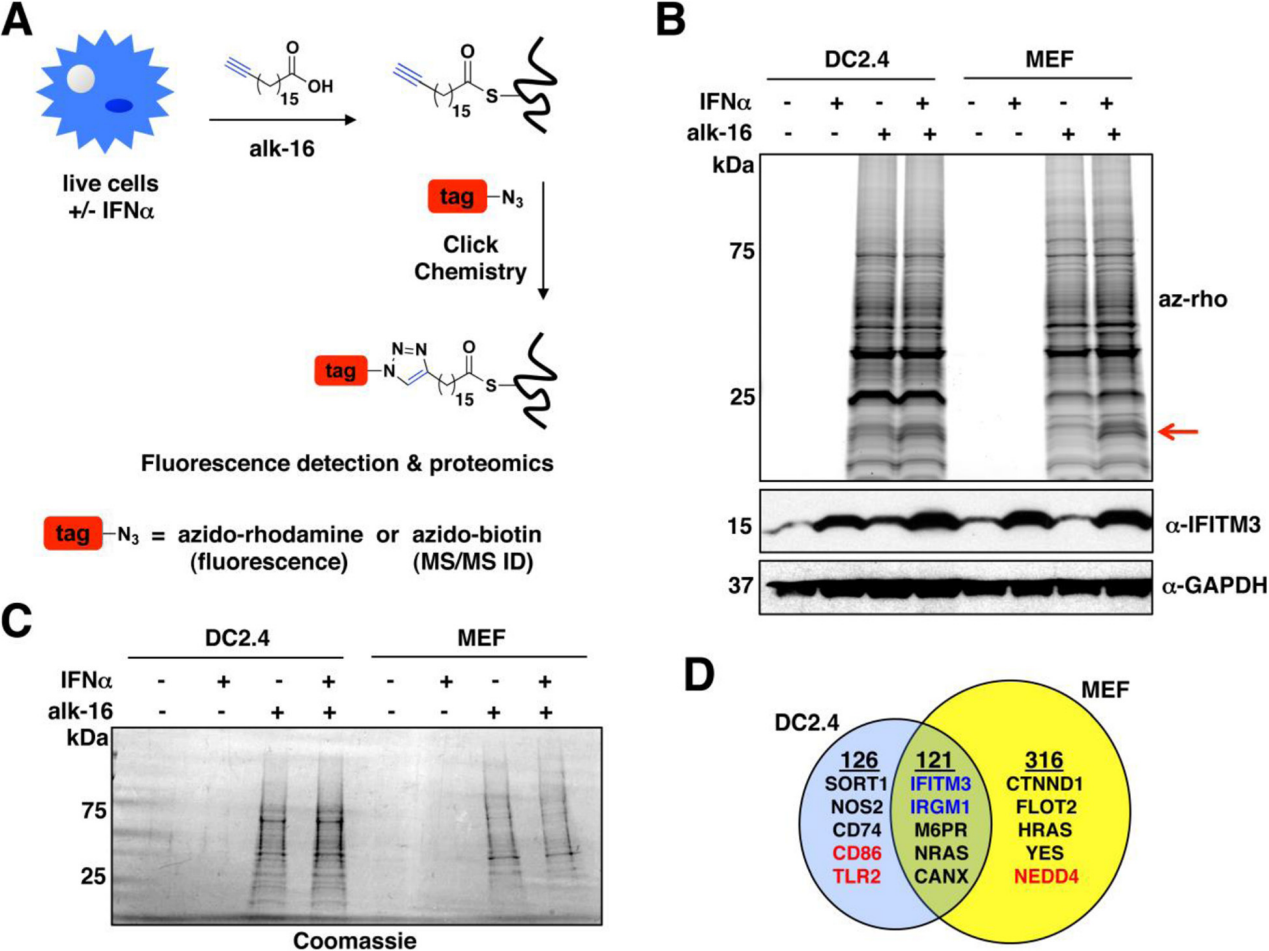
**Identification of palmitoylated proteins in DC2.4 cells and MEFs. A)** Schematic depicting alk-16 chemical reporter metabolic labeling of live cells and subsequent reaction of cell lysates with detection tags for fluorescence visualization of palmitoylated proteins or affinity purification and proteomic identification of palmitoylated proteins. **B, C)** Mock treated or IFNα treated DC2.4 cells and MEFS were labeled with alk-16 or DMSO as a control. Lysates were subjected to click chemistry with azido-rhodamine (az-rho) **(B)** or azido-biotin **(C)**. **B)** Proteins were separated by SDS-PAGE for fluorescence gel scanning to visualize palmitoylome profiles. Anti-IFITM3 Western blotting served as a control for the activity of IFNα and anti-GAPDH blotting served as a loading control. The red arrow points to the most prominent change in the banding pattern when comparing mock and IFNα treated samples. **C)** Alk-16/azido-biotin labeled proteins enriched using streptavidin agarose were separated by SDS-PAGE, and were stained with coomassie blue for visualization. Each lane was cut into slices for trypsin digestion and extraction of peptides for MS/MS identification. **D)** Venn diagram representing proteins identified in **C**. After subtracting proteins identified in control lanes, 237 DC2.4 proteins and 438 MEF proteins were considered putative palmitoylated proteins. The majority of identified proteins were known to be palmitoylated, including most proteins identified in both cell types and also many of those specifically identified in either DC2.4 cells or MEFs (examples of each are shown in black text). The known IFN-induced and palmitoylated proteins IFITM3 and IRGM1 (blue text) were identified as upregulated in both IFNα-treated DC2.4 cells and MEFs. CD86 and TLR2 are example immunomodulatory proteins specifically identified in DC2.4 cells, while NEDD4 is an example protein specifically identified in MEFs (red text). DMSO, dimethyl sulfoxide; IFITM3, interferon (IFN)-induced transmembrane protein 3; IRFM1, immunity related GTPase M1; MEFs, murine embryonic fibroblasts; MS/MS, tandem mass spectrometry; TLR2, Toll-like receptor 2.

Protein palmitoylation is the addition of a 16-carbon fatty acid primarily to cysteines via a thioester linkage (termed S-palmitoylation) [1]. This form of fatty acylation targets cytoplasmic proteins to membranes, but can also occur on transmembrane proteins where it often affects protein localization or stability [1-3]. Identification and characterization of palmitoylated proteins has traditionally been encumbered because of insensitive detection methods, the lack of a consensus amino acid motif for bioinformatics prediction of palmitoylation sites, a lack of antibodies for detecting palmitoylation and challenges in detecting palmitoylated peptides with standard mass spectrometry techniques [4,5]. To overcome these difficulties, non-radioactive chemical tools and methodologies have been recently developed that are advancing the study of protein palmitoylation [6-11]*.* An analogue of palmitic acid possessing a terminal alkyne group (alk-16, Figure 1A) is incorporated by cells onto proteins at sites of palmitoylation [6]. The alkynyl group allows a targeted reaction with azide-functionalized detection tags via the copper-catalyzed azide-alkyne cycloaddition reaction commonly termed click chemistry (Figure 1A). Azido-rhodamine (az-rho) is a detection tag that can be used for visualization, while azido-azo-biotin (az-biotin) allows selective retrieval of alk-16-labeled proteins using streptavidin-coated agarose [7,8,12]. We, and others, have previously used, and continue to apply these tools for enhancing our understanding of regulation of immune responses by lipid post-translational modifications (PTMs) [7,11,13-17].

Of particular importance, the aforementioned chemical tools allowed the identification of palmitoylation-dependent antiviral activity of the interferon (IFN)-induced transmembrane protein 3 (IFITM3) [7]. In the present study, we performed an analysis of palmitoylated proteins in a murine antigen presenting dendritic cell line (DC2.4) in comparison with murine embryonic fibroblasts (MEFs), both at steady state and after IFNα treatment. Known lipidated and IFN-induced proteins, IFITM3, bone marrow stromal antigen 2 (BST2) and immunity related GTPase M1 (IRGM1), were detected in both cell types after IFNα treatment. We also detected novel palmitoylated proteins expressed at steady state, and validated several of these proteins, including T-lymphocyte activation antigen CD86 and Toll-like receptor 2 (TLR2) in dendritic cells (DCs), and E3 ubiquitin-protein ligase NEDD4 in MEFs. Given the complete novelty of the discovery of a lipid modification occurring on a member of the extensively studied TLR family, we chose to focus further on determining the effects of palmitoylation on TLR2.

TLRs are critical for the cellular recognition of most pathogens [18]. Their detection of microbial products results in activation of the transcription factor NF-κB and production of inflammatory cytokines and other mediators of the immune response [18]. At least 10 human and 11 mouse TLRs have now been identified, which recognize distinct sets of pathogen-associated molecules [19]. TLR2 is expressed primarily on myelomonocytic cells including antigen presenting cells, such as DCs and macrophages [20]. TLR2 detects the widest range of microbial products among the TLRs, including lipomannan from mycobacteria, zymosan from yeast and bacterial lipopeptides typified by the PamCSK synthetic lipopeptides [18,21]. As such, TLR2 knockout mice and humans with deleterious TLR2 polymorphisms are more susceptible to multiple pathogens [22-26]. Thus, TLR2 is a critical component of the innate immune system, and a better understanding of its post-translational regulation may prove useful in our defense against pathogenic organisms.

## Results and discussion

### Visualization of palmitoylated proteins in MEFs and DC2.4 cells

Having made the previous discovery of the critical role of palmitoylation of IFITMs in the innate antiviral immune response [7,14,15], we sought to determine whether any additional IFN-induced proteins are regulated by palmitoylation. For our experiments, we chose to use murine antigen presenting cells (DC2.4) and MEFs because these cell lines are responsive to type I IFNs [14], are amenable to labeling with the alk-16 reporter of protein palmitoylation [6,14] and serve as a control for one another in that type I IFN should induce a similar set of proteins in both cell types. Additionally, this analysis would provide a valuable comparison of the general palmitoylomes of two cell types (myeloid and non-myeloid) with unique functions. DC2.4 cells and MEFs were either left untreated or were treated with IFNα for four hours prior to metabolic labeling with alk-16 in the presence or absence of IFNα for an additional two hours. Cell lysates were reacted with az-rho or az-biotin via click chemistry (Figure 1A). Az-rho-labeled proteins were separated by SDS-PAGE and visualized by fluorescence gel scanning (Figure 1B). Anti-IFITM3 and anti-GAPDH blotting were used as controls for the activity of IFNα on these cells and for loading, respectively. The fluorescent profiles of the two cell types did not indicate that drastic changes to palmitoylation occur upon IFNα treatment. However, one specific band could be seen uniquely in the IFNα-treated samples, appearing at approximately 15 kDa, potentially corresponding to the molecular weight of the IFITMs (Figure 1B).

### Mass spectrometric identification of palmitoylated proteins in MEFs and DC2.4 cells

Given that differences induced by IFNα could possibly be masked by highly abundant proteins within the fluorescent palmitoylome profiles observed in Figure 1B, we next utilized the fraction of our samples that had been reacted with az-biotin in order to affinity purify alk-16-labeled palmitoylated proteins using streptavidin-coated agarose. These purified proteins were separated by SDS-PAGE (Figure 1C) and subjected to in-gel trypsin digestion. Extracted peptides were then identified by tandem mass spectrometry (MS/MS) analysis and assigned to proteins using Mascot software. After subtraction of proteins identified in the control lanes as described in the Methods Section and eliminating proteins with fewer than three spectral counts, a total of 247 proteins were identified from the DC2.4 samples and 437 proteins were identified from the MEF samples, indicating that there is a large difference in the repertoire of palmitoylated proteins expressed by these different cell types (see Additional file 1: Table S1, S2, Figure 1D). Of the proteins identified in DC2.4 cells, 76% had been previously identified as palmitoylated proteins in our past analysis of this cell line, and by others in proteomic studies of other cell types [7,16,17,27-31] (see Additional file 1: Table S1). Of the proteins identified in MEFs, which had not been palmitoylome profiled before, 64% were previously reported to be palmitoylated, providing strong validation of our methodology (see Additional file 1: Table S2).

Analysis of several known palmitoylated proteins that were identified uniquely in only one of the two cell types would suggest that their differential detection is due to cell type-specific expression differences. For example, sortilin-1 (SORT1), inducible nitric oxide synthase (NOS2) and HLA class II histocompatibility antigen gamma chain (CD74) were uniquely detected in DC2.4 cells, consistent with published data curated by BioGPS showing that murine DCs express these genes while MEFs do not [32-34] (Figure 1D). Conversely, catenin delta (CTNND1), flotillin-2 (FLOT2), GTPase HRAS and tyrosine-protein kinase YES were detected in MEFs, but not in DCs (Figure 1D), again corresponding to cell type-specific expression patterns [32-34]. Further supporting the assertion that the observed differences in the proteins detected are not due to differences in the ability of the two cell types to palmitoylate different subsets of proteins, BioGPS was also utilized to examine the MEF and DC expression patterns for the DHHC palmitoyltransferases (hereafter referred to as DHHCs), the enzyme family responsible for the majority of S-palmitoylation in cells [35,36]. Indeed, murine MEFs and DCs each express nearly all of the DHHCs, with the exception of DHHCs 11 and 19 which are testis-specific and DHHC22 which is brain-specific [32-34].

### Bioinformatic classification of alk-16-labeled proteins commonly identified in both MEFs and DC2.4 cells

We identified 121 alk-16-labeled proteins that were common to both DC2.4 cells and MEFs (listed in Additional file 1: Table S3), suggesting that these proteins may perform fundamental cellular functions. These included critical cellular proteins such as calnexin (CANX), GTPase NRAS and the cation-dependent mannose-6-phosphate receptor (M6PR) (Figure 1D). Indeed, exploring the classification of these 121 proteins shows that proteins with enzymatic activity constituted 40% of the 121. Transporters and ion channels formed another 20%. An additional 37% constituted structural proteins and those in ubiquination and glycosolation processes, with the remaining 3% being cellular receptors and transcription regulators (see Additional file 2: Figure S2A). Enrichment analysis using Ingenuity defined pathways recapitulated that palmitoylation is critical for a multitude of signaling pathways, including those downstream of ephrin receptors and integrins, as well as axonal guidance signaling (see Additional file 1: Table S4). Similarly, pathways centering on the cellular morphology-controlling small GTPases, RAC1 and/or RHOA, involving multiple palmitoylated proteins were identified (see Additional file 1: Table S4).

On a more global level, network analysis of the 121 shared palmitoylated proteins demonstrated that the MYC proto-oncogene protein and the cellular tumor antigen p53 are upstream of 35 (29%) of these proteins, which is more than would be expected by chance (*P* <0.001, Additional file 2: Figure S2B). Several DHHCs have been discovered to be misregulated in various tumors, but their contribution to tumorigenesis is currently unclear [37]. Palmitoylated MYC and p53 effector proteins may offer candidates for future experiments determining the role of specific DHHCs and palmitoylated proteins in cancer progression. Taken together, a multitude of coherent pathways are regulated by palmitoylation in both DCs and MEFs, and these data add to our growing appreciation of the roles palmitoylation plays in regulating a myriad of basic cellular processes.

### IFN-induced fatty acylated proteins

We also identified proteins present only in the IFNα-treated samples or that had peptide peak area measurements that were increased in the IFNα-treated samples by at least 2.5 fold. Since type I IFNs generally induce a common set of antiviral proteins even in distinct cell types, we reasoned that true IFN-induced palmitoylated proteins should be present in the MS/MS results of both DC2.4 cells and MEFs. With this additional criterion, we were left with only three proteins enriched in the IFNα-treated samples in both cell types: IFITM3, IRGM1 and BST2, also known as tetherin. Indeed, each of these proteins is known to be IFN-inducible, and IFITM3 serves as a control since we expected to identify this protein based on our past work [7]. Palmitoylation of IRGM1 was verified in our laboratory (see Additional file 2: Figure S1), and was also recently reported to be important for its membrane localization and ability to affect mitochondria membrane dynamics [38]. Interestingly, past palmitoylome profiling studies of murine T cells [17] and human B cells [16] also identified BST2 as a putative palmitoylated protein. Although BST2 palmitoylation has not yet been validated by visualization methods, the study performed in murine T cells found that mass spectrometric detection of alk-16-labeled BST2 was diminished upon sample treatment with hydroxylamine, which cleaves thioester bonds characteristic of palmitoylation [17]. Likewise, the work identifying BST2 in B cells utilized acyl-biotin exchange chemistry, which allows specific enrichment of S-acylated proteins [16]. BST2 is also well characterized to be lipidated with a GPI anchor [39], which could incorporate long chain fatty acids such as alk-16, although previous alk-16 proteomic profiling studies have not been significantly contaminated with GPI anchored proteins, arguing against this possibility [7,9,11,17,27]. Mutation of individual residues within BST2 will be necessary to determine the precise location and type of lipidation reported by alk-16 labeling. Nonetheless, our data confirm that the IFN-induced effector proteins, IFITM3, IRGM1 and BST2, are each lipid-modified.

### Novel palmitoylated proteins identified in MEFs and DC2.4 cells

**Figure 2:**
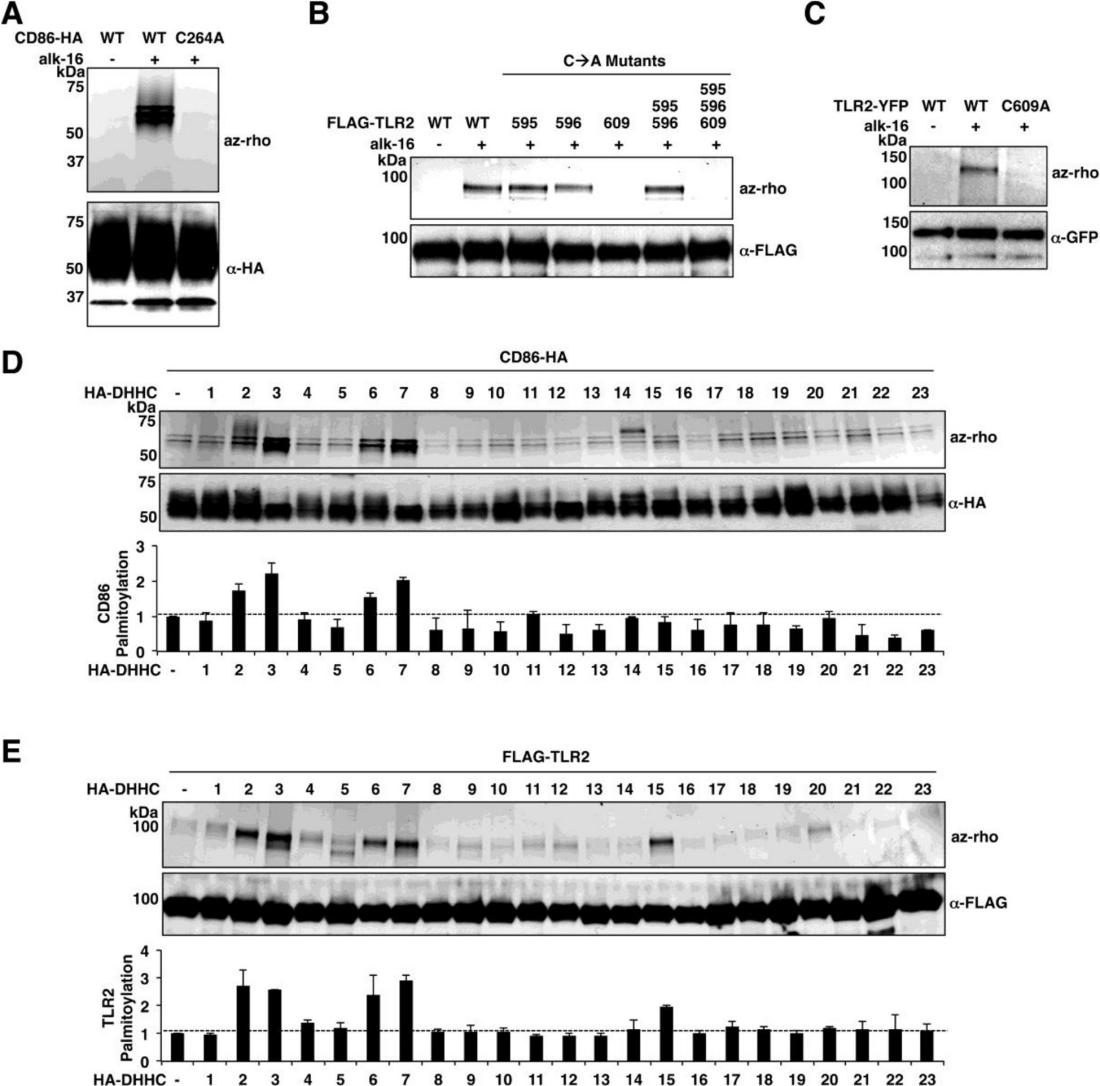
**Confirmation of the S-palmitoylation of immunomodulatory proteins, CD86 and TLR2. A-C)** Validation and mapping of CD86 and TLR2 palmitoylation. HEK293T cells were transfected with the indicated CD86-HA **(A)**, FLAG-TLR2 **(B)**, or TLR2-YFP **(C)** constructs. Transfected cells were labeled with 50 uM alk-16 or DMSO as a control for one hour. Cell lysates were subjected to immunoprecipitation for the respective epitope tag or fluorescent protein. Immunoprecipitates were reacted with azido-rhodamine (az-rho) for visualization of palmitoylation by fluorescent gel scanning. Western blotting was performed to confirm comparable protein loading. **D,E)** Overexpression screens to identifiy DHHCs capable of modifying CD86-HA and FLAG-TLR2. HEK293T cells were co-transfected with HA-tagged DHHCs 1 to 23 or GST as a control and either CD86-HA **(D)** or FLAG-TLR2 **(E)**. Immunoprecipitated proteins were treated and visualized as in **A-C**. Western blotting was performed to confirm comparable protein loading. Bar graphs represent average quantified fluorescence intensities from at least three identical experiments normalized to their respective western blots. Values for CD86 or TLR2 co-transfected with GST control were set to 1. DHHC constructs are labeled according to previously used DHHC nomenclature in order to be consistent with past studies using these constructs. Importantly, DHHCs 2, 3, 6, 7 and 15 are the same in both the previous and modern nomenclature. Note that in **D**, DHHC14 itself is visible in the fluorescent gel scan of this region. The expression levels of the individual DHHCs obtained in a representative experiment are shown in Additional file 2: Figure S6. DMSO, dimethyl sulfoxide; TLR2 Toll-like receptor 2.

Our work identified 168 proteins in MEFs and 59 proteins in DC2.4 cells that had not been previously reported to be palmitoylated in past proteomic studies (see Additional file 1: Table S1, S2). Many of these proteins were found in only one of the two cell types. For example, we observed the E3 ubiquitin-protein ligase NEDD4 specifically in MEFs (Figure 1D), and confirmed its palmitoylation, thereby identifying an entirely new modification with potential regulatory activity on this extensively studied and critically important protein [40,41] (see Additional file 2: Figure S3). Of particular interest to our laboratory were two proteins uniquely identified in DC2.4 cells that are essential to immune responses, CD86 and TLR2. CD86 is expressed on the surface of mature DCs and provides a costimulatory signal to T cells that is necessary for their proper activation [42]. We thus sought to confirm CD86 palmitoylation and map its site of modification. Utilizing a C-terminally HA-tagged construct of murine CD86 (CD86-HA), we were able to validate our MS/MS results by demonstrating robust labeling of CD86-HA with alk-16 (Figure 2A). Interestingly, palmitoylated CD86-HA was observed as multiple high MW bands indicating that its glycosylated forms are palmitoylated [43], while its non-glycosylated form at the expected molecular weight of 35 kDa was not modified, potentially suggesting sequential modification (Figure 2A). Mutation of Cys-264 to Ala at the cytoplasmic edge of the CD86 transmembrane domain resulted in a complete loss of alk-16 labeling, implicating Cys-264 as the primary site of CD86 palmitoylation (Figure 2A). CD86 has homology to, and a partially redundant function with, T-lymphocyte activation antigen CD80 [44]. We thus generated a CD80-HA construct and also confirmed its palmitoylation (see Additional file 2: Figure S4), thereby demonstrating a conservation of this PTM on the CD80 and CD86 costimulatory molecules.

We similarly confirmed TLR2 palmitoylation utilizing a previously generated construct in which the human TLR2 ER signal sequence was replaced by an artificial signal sequence followed by a FLAG epitope tag [45,46]. Indeed, FLAG-TLR2 was readily labeled by alk-16 (Figure 2B). Two cytoplasmic-facing cysteines within TLR2, Cys-595 and Cys-596, were predicted by the CSS-Palm version 3.0 palmitoylation site prediction program [47] to be highly probable sites of palmitoylation. Generating Cys to Ala mutations at these positions and at the nearby Cys-609 allowed for the demonstration that palmitoylation is lost only when Cys-609 is mutated (Figure 2B). Thus, while bioinformatic methods may provide candidate sites of modification, the study of protein palmitoylation requires empirical validation. We further confirmed that Cys-609 is the primary site of human TLR2 S-palmitoylation using a TLR2 construct with its natural ER signal sequence and a C-terminal YFP tag [48,49] (Figure 2C), and also verified that murine TLR2 is S-palmitoylated on the same residue (see Additional file 2: Figure S5).

To determine the extent to which palmitoylation is conserved on TLRs other than TLR2, we tested a panel of human TLRs 1 to 10 for alk-16 labeling in HEK293T cells. While the expression levels of the different TLRs varied, TLR2, TLR5 and TLR10 clearly showed palmitoylation signals above background bands (see Additional file 2: Figure S5). Interestingly, TLR10, which physically interacts with TLR2 and has been associated with regulating TLR2 responses (43, 44), had the strongest palmitoylation signal relative to its total protein level (see Additional file 2: Figure S5). These findings reveal that palmitoylation is at least partially conserved on a subset of the human TLRs. These data also confirm that palmitoylation of transmembrane proteins occurs with specificity that is not currently predictable by sequence analysis or by homology with known palmitoylated proteins.

### Palmitoylation of CD86 and TLR2 can be increased by specific DHHCs

In order to examine whether or not S-palmitoylation of CD86 and TLR2 could be installed by specific DHHCs, we performed overexpression screens of each of the DHHCs with CD86 and TLR2. This method is commonly used to identify DHHCs capable of modifying individual protein substrates [35,50-53]. CD86-HA S-palmitoylation was increased by DHHCs 2, 3, 6 and 7 (Figure 2D, Additional file 2: Figure S7). FLAG-TLR2 S-palmitoylation was similarly enhanced by the DHHCs 2, 3, 6 and 7 (Figure 2E, Additional file 2: Figure S7). Since the palmitoylation sites of CD86 and TLR2 are both at the cytoplasmic edge of their respective transmembrane domains, this may suggest that this subset of DHHCs is particularly adept at modifying cysteines adjacent to membranes. However, unlike CD86, palmitoylation of TLR2 was also increased by DHHC15, demonstrating substrate specificity for individual DHHCs (Figure 2E). Overall, these experiments further validate that CD86 and TLR2 are S-palmitoylated proteins whose modification can be mediated enzymatically by specific DHHCs.

### Palmitoylation is required for complete activation of NF-κB by TLR2

**Figure 3:**
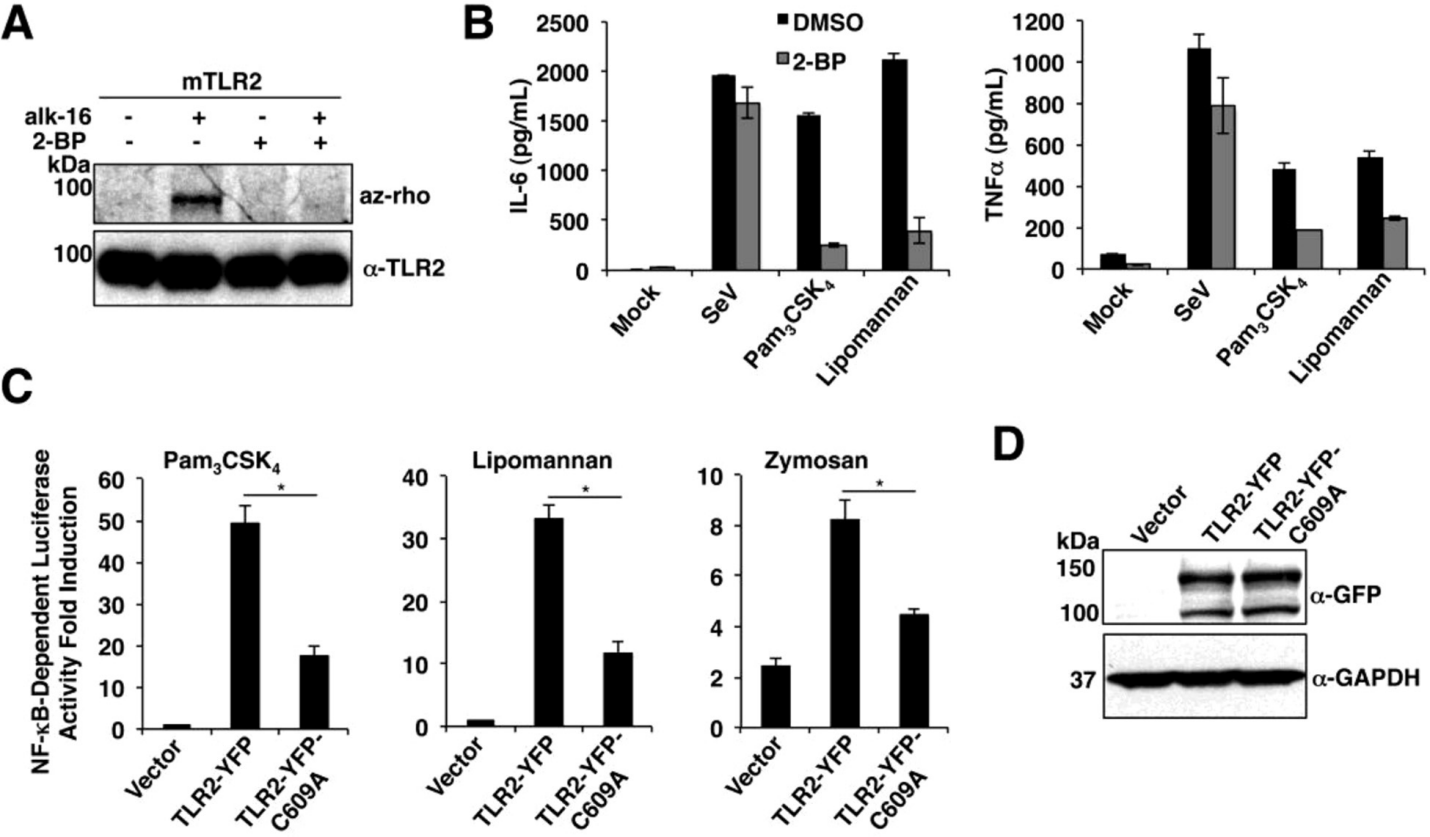
**TLR2 S-palmitoylation is necessary for NF-kB-dependent gene induction and cytokine production in response to TLR2 microbial ligands. A)** TLR2 S-palmitoylation is inhibited by 2-BP in BMDCs. Cells were treated with 100 uM 2-BP or an equivalent volume of DMSO for eight hours prior to labeling for one hour with 50 uM alk-16. TLR2 was immunoprecipitated from cell lysates and reacted with az-rho for fluorescence visualization of palmitoylation. Western blotting with anti-TLR2 antibodies was performed to confirm comparable protein loading. **B)** Cytokine secretion in reponse to TLR2 ligands is inhibited by 2-BP in BMDCs. Cells were treated with 100 uM 2-BP or an equivalent volume of DMSO for eight hours. TLR2 ligands, Pam_3_CSK_4_ (2 ug/mL) or lipomannan (4 ug/mL), or Sendai virus (SeV) (MOI 5), were added to the cellular media for an additional six hours. IL-6 and TNFα levels in cellular supernatants were measured by ELISA. Results in **A,B** are representative of three experiments. Error bars in **B** are the standard deviation of triplicate samples. **C)** Palmitoylation-deficient TLR2 is less able than WT to induce NF-κB-dependent gene expression in response to microbial ligands. HEK293T cells were co-transfected with the indicated plasmids along with a reporter construct expressing NF-κB-driven firefly luciferase and a plasmid constitutively expressing renilla luciferase for normalization. Cells were mock treated or treated for eight hours with Pam_3_CSK_4_ (2 ug/mL), lipomannan (4 ug/mL) or zymosan (10 ug/mL), and luciferase activity was measured in cell lysates. Results are presented as fold induction over mock treated samples. **D)** Western blots confirming similar expression of TLR2-YFP and TLR2-C609A-YFP in experiments performed in **C**. Results in **C, D** are representative of at least five experiments. Error bars in **C** represent standard deviation of triplicate samples. *P <0.001 by Student’s *t*-test. az-rho, azido-rhodamne; BMDCs, bone marrow dendritic cells; DMSO dimethyl sulfoxide; TLR, Toll-like receptor; 2-BP, 2-bromopalmitate.

Given our laboratory’s focus on innate immunity, we chose to further investigate the effects of S-palmitoylation on the activity of TLR2 in primary cultured DCs. For these experiments, we utilized a commonly used covalent inhibitor of DHHCs, 2-bromopalmitate (2-BP) [54]. We found that 2-BP treatment of murine bone marrow-derived (BM) DCs resulted in a loss of the endogenous TLR2 S-palmitoylation signal (Figure 3A). Concomitantly, 2-BP-treated BMDCs lost the ability to produce high levels of the pro-inflammatory cytokines IL-6 and TNFα in response to Pam_3_CSK_4_ and lipomannan, which are microbe-based ligands specifically detected by TLR2 (Figure 3B). Since prolonged exposure to 2-BP can result in off-target effects and cellular toxicity [55,56], we also employed Sendai virus, a TLR-independent activator of cytoplasmic RIG-I-like receptors, as a control [57-61]. Sendai virus induced high levels of IL-6 and TNFα secretion even in the presence of 2-BP (although TNFα was partially affected), indicating that the effects of 2-BP were largely specific to the TLR2 pathway (Figure 3B). Additionally, we did not observe overt cellular toxicity upon 2-BP treatment in the time period examined, as judged by Trypan blue exclusion. Treatment with 2-BP also inhibited lipomannan-induced IL-6 and TNFα secretion from primary human monocyte-derived DCs (see Additional file 2: Figure S8). Thus, the inhibition of cellular palmitoylation by 2-BP leads to an impaired TLR2-dependent proinflammatory cytokine response by DCs.

We next measured the ability of TLR2-YFP and TLR2-C609A-YFP to stimulate NF-κB-dependent gene expression in response to microbial ligands. TLR2-C609A-YFP was significantly less able than TLR2-YFP to induce NF-κB-dependent luciferase production in response to Pam_3_CSK_4_, lipomannan and zymosan (Figure 3C). Levels of WT and mutant TLR2 were comparable in these experiments as measured by Western blotting (Figure 3D). Similar results demonstrating a defect in the induction of NF-κB-dependent luciferase activity by the palmitoylation-deficient TLR2-C609A mutant were also obtained when utilizing FLAG-tagged constructs, and further demonstrated that this decrease in activity is specific for mutation of the Cys-609 palmitoylation site since a Cys-596 to Ala mutant possessed activity similar to WT FLAG-TLR2 (see Additional file 2: Figure S9). In sum, our data demonstrate that the TLR2 Cys-609 S-palmitoylation site is required for proper activation of NF-κB in response to microbial ligands.

### S-pamitoylation of TLR2 promotes its cell surface localization

**Figure 4:**
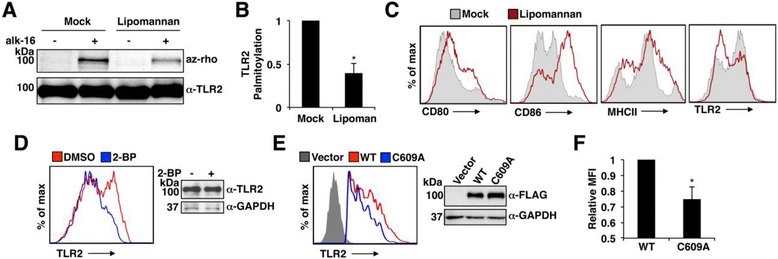
**S-palmitoylation promotes TLR2 cell surface expression. A-C)** BMDCs were mock treated or treated with 4 ug/mL lipomannan for 24 hours. **A)** Cells were labeled with 50 uM alk-16 for one hour. Immunoprecipitated TLR2 was reacted with az-rho for visualization of palmitoylation. Anti-TLR2 Western blotting confirmed comparable protein loading. **B)** Fluorescent gel scans from four experiments as in **A** were quantified and normalized to their respective Western blots. Values for lipomannan were normalized relative to a value set to 1 for mock, and were averaged. The error bar represents the standard deviation of four experiments. *P <0.001 by Student’s *t*-test. **C)** Cells were stained with antibodies against the indicated surface proteins and analyzed by flow cytometry. Results in **C** are representative of at least three experiments. **D)** BMDCs were treated with 100 uM 2-BP or solvent control for 12 hours, and stained with anti-TLR2 antibody for flow cytometry analysis of TLR2 surface levels, or were lysed and subjected to anti-TLR2 Western blotting to examine total TLR2 levels. Anti-GAPDH blotting served as a loading control. Results in **D** are representative of at least three similar experiments. **E, F)** MEFs were transfected with plasmids expressing murine FLAG-TLR2 (WT), FLAG-TLR2-C609A (C609A), or vector control. **E)** Cells were subjected to anti-TLR2 staining for flow cytometry analysis of TLR2 surface expression or were lysed for western blotting with anti-FLAG antibodies for comparing total TLR2 protein levels. Anti-GAPDH Western blotting was performed as a loading control. **F)** Flow cytometry results from three experiments as in **E** were quantified in terms of mean fluorescence intensity (MFI). Values for C609A were normalized relative to a value set to 1 for WT, and were averaged. The error bar represents the standard deviation of three experiments. *P <0.01 by Student’s *t*-test. az-rho, azido-rhodamne; BMDCs, bone marrow dendritic cells; MEFs, murine embryonic fibroblasts; TLR, Toll-like receptor.

We next examined the effect of endogenous TLR2 ligation on its S-palmitoylation status. We observed that TLR2 palmitoylation is decreased upon stimulation of murine BMDCs with lipomannan (Figure 4A,B). This palmitoylation decrease correlates with a decrease in TLR2 surface staining, while surface staining of costimulatory molecules and MHC II increased as expected for activated DCs (Figure 4C). This finding is also consistent with past observations of TLR2 downregulation at the cell surface upon binding of its ligands [62-64]. The correlation between decreased TLR2 S-palmitoylation and decreased cell surface levels suggested that, perhaps, S-palmitoylation affects TLR2 localization. Indeed, inhibition of palmitoylation with 2-BP decreased endogenous TLR2 surface staining of BMDCs, while overall cellular levels of TLR2 remained similar (Figure 4D). In accord with these results, overexpressed murine FLAG-TLR2-C609A showed a subtle, but reproducible, decrease in cell surface staining when compared to WT FLAG-TLR2 (Figure 4E), with their mean fluorescence intensities differing by approximately 25% on average (Figure 4F). Importantly, achieving robust cell surface staining of FLAG-TLR2 constructs required transfection of five to ten times more DNA than was required for activity assays, perhaps suggesting that the forced overexpression of the WT and C609A TLR2 constructs results in underestimation of their cell surface differences. We further confirmed that overall protein levels and the stability of TLR2 are not affected by palmitoylation by performing pulse-chase analysis of FLAG-TLR2 and FLAG-TLR2-C609A, which demonstrated that the decay of the WT and mutant proteins was similar (see Additional file 2: Figure S10). Overall, we conclude that S-palmitoylation positively regulates TLR2 activity, at least in part, by promoting its trafficking to the cell surface.

## Conclusions

The development of chemical proteomic methods to identify palmitoylated proteins has led to a tremendous increase in our appreciation of the numerous ways in which palmitoylation regulates key cellular processes and can control cell type-specific pathways [7,9,11,16,17,28-31,65]. We previously reported the identification of 157 palmitoylated proteins in DC2.4 cells that included proteins critical to antiviral immune responses [7]. However, the absence of several expected palmitoylated proteins from our MS/MS identification led us to hypothesize that additional unknown palmitoylated proteins also remained to be identified in this cell type [13]. Indeed, refinements to our palmitoylated protein enrichment protocol that included increased incubation time with streptavidin agarose and repeated washing steps, along with updates to our mass spectrometry capabilities, have led to a deeper coverage of the DC2.4 palmitoylome (Figure 1 and Additional file 1: Table S1). In comparison to the palmitoylome of MEFs, we identified novel putative palmitoylated proteins that play a role in the immune functions of DCs. Following up on several of these proteins, we were able to validate palmitoylation of the CD80 and CD86 costimulatory molecules (Figure 2A and Additional file 2: Figure S4) as well as TLR2 (Figure 2B,C and Additional file 2: Figure S5). Interestingly, our proteomic results indicated that MEFs express a wider diversity of palmitoylated proteins than DCs (Figure 1D and Additional file 1: Table S1, S2), and include many proteins, such as NEDD4, that should lead to exciting follow up studies.

Our discovery of CD80, CD86 and TLR2 palmitoylation in DCs reveals novel mechanisms of immune regulation, and underscores the value of examining palmitoylation in diverse cells types. CD80 and CD86 are essential for immune responses to most pathogens as well as for elimination of tumor cells by the immune system [66]. These costimulatory molecules provide a second signal for T-cell activation in addition to MHC/peptide complexes presented on the surface of mature DCs [66]. Future studies will aim to determine whether palmitoylation is involved in the trafficking of these molecules to the DC surface, their subsequent clustering at the immunological synapse and their impact on immune function.

Here, we specifically followed up on the role of S-palmitoylation in regulating TLR2 activity. Indeed, the response to all microbial ligands of TLR2 that were tested was decreased in the absence of TLR2 S-palmitoylation, indicating that this lipid modification is a positive regulator of TLR2 activity (Figure 3 and Additional file 2: Figure S8, S9). S-palmitoylation is necessary for complete localization of TLR2 at the cell surface, and thus likely affects its interactions with its ligands (Figure 4D-F). While an effect of palmitoylation on the cell surface expression of multiple other proteins has been previously reported [3], it is interesting to note that although CD86 and TLR2 are both S-palmitoylated at membrane-proximal cysteines (Figure 2A-C) by a similar subset of DHHCs (Figure 2D,E), their cell surface localization is inversely regulated during DC activation (Figure 4B). This reinforces the commonly observed theme that palmitoylation can have unpredictable effects depending on the protein context in which it occurs [1,3,67]. Future analyses of how S-palmitoylation of additional immune receptors controls their localization, interactions and signaling will be an exciting area of future study, as will the examination of potential subversion of TLR2 S-palmitoylation by pathogens. Importantly, the identification of S-palmitoylation of TLRs suggests that this reversible form of protein lipidation [68] may be targeted in the future to manipulate beneficial and non-beneficial TLR2-mediated immune responses.

## Methods

### Click chemistry, fluorescence gel scanning, and proteomic identification of palmitoylated proteins

Click chemistry was performed as previously described [6-8,69]. In short, immunoprecipitated proteins or cell lysates were reacted for one hour at room temperature with 100 μM azido-rhodamine [6] or azido-azo-biotin [12], 1 mM tris(2-carboxyethyl)phosphine hydrochloride (TCEP), 100 μM tris[(1-benzyl-1*H*-1,2,3-triazol-4-yl)methyl]amine (TBTA), and 1 mM CuSO_4_ · 5H_2_O. In-gel fluorescence scanning was performed using a Typhoon 9400 imager (Amersham Biosciences, Amersham, United Kingdom) (excitation 532 nm, 580 nm detection filter). Enrichment of alk-16-labeled proteins was performed according to a standard protocol [7-9] with minor adjustments in the amount of protein and buffers used and the number of washing steps, in order to increase specificity and recovery of labeled proteins. Proteins in cell lysates reacted with azido-azo-biotin were methanol precipitated at −20°C overnight, washed with ice-cold methanol three times, dried, and resolubilized in SDS buffer (4% SDS, 150 mM NaCl, 50 mM triethanolamine, pH 7.4). A total of 2.5 mg of the resolubilized protein in 1 mL was diluted to 15 mL with PBS containing 1% Brij-97 and was incubated with 100 μL of pre-washed high-capacity streptavidin agarose (Thermo Scientific, Waltham, Massachusetts, United States) at 4°C on a nutating mixer overnight. The streptavidin agarose was then washed five times with 15 mL PBS containing 1% SDS followed by three washes with 15 mL PBS and two washes with 250 mM ammonium bicarbonate. Cysteines were then capped with iodoacetamide, followed by three additional washes with 250 mM ammonium bicarbonate. Proteins were eluted from the agarose three times using sodium dithionite, and concentrated using low molecular weight Centricons (Millipore, Billerica, Massachusetts, United States) as previously described [8]. Proteins were then separated by SDS-PAGE for in-gel digestion, peptide extraction and MS/MS analysis. MS/MS was performed by the Rockefeller University Proteomics Resource Center on a Thermo LTQ-Orbitrap mass spectrometer that had undergone recent professional tuning and alignment, and that was equipped with a Dionex 3000 capillary/nano HPLC. MS/MS spectra of peptides were analyzed against the mouse protein database using the Mascot version 2.3 search tool. Data from samples analyzed by Mascot were compared with ProteomeDiscoverer software (Thermo Scientific) utilizing a cutoff score of 40 and a requirement of two peptides per protein. Data were then further manually analyzed by eliminating proteins that were identified with less than a 20-fold detection increase over the controls based on the ProteomeDiscoverer software peptide peak area measurement, and by eliminating proteins with a sum of less than three spectral counts in the alk-16-treated samples. Proteomic lists from other studies were compared with this data set using gene symbols obtained, if necessary, by converting published identifiers into gene symbols using the UniProt database along with the DAVID Gene ID Conversion Tool (National Institute of Allergy and Infectious Diseases, National Insttutes of Health (NIAID, NIH)).

### Pathway enrichment and network analysis

Protein data from DC2.4 and MEF cells with suitable quality as described above were standardized using GenPept accession numbers. Overlapping proteins were subsequently ported into Ingenuity IPA software [70]. Canonical pathway enrichment was performed using IPA defined classes and corrected for multiplicity using the Benjamini-Hochberg method. Network analysis and subsequent diagrams were generated using only direct associations among the proteins. *P*-values for upstream analysis were calculated using the Fisher’s exact test.

### DNA constructs and luciferase assays

TLR2-YFP, pCDNA3-YFP and fluorescent protein-tagged constructs for the other human TLRs were obtained from Addgene (plasmids 13014, 13033, 13016, 13641, 13018, 13019, 13020, 13022, 13024, 13642, and 13643, deposited by Doug Golenbock). The human FLAG-TLR2 construct was also obtained from Addgene (plasmid 13082, deposited by Ruslan Medzhitov), as was the mouse FLAG-TLR2 construct (plasmid 12291 deposited by Bruce Beutler [71]). CD80 and CD86 coding sequences were amplified by PCR from DC2.4 cell cDNA incorporating a C-terminal HA tag, as well as EcoRI and BglII restriction sites for insertion into the pCAGGS expression vector. IRGM1 coding sequence was amplified by PCR from DC2.4 cell cDNA incorporating SalI and XhoI restriction sites for in-frame insertion into the pCMV-HA expression vector. The QuikChange Multi-Site Directed Mutagenesis Kit from Stratagene (La Jolla, California, United States) was used for cysteine mutagenesis. HA-tagged DHHCs and GST control plasmids were kindly provided by Masaki Fukata (NIPS, Okazaki, Japan). For luciferase assays, a NF-kB-dependent firefly luciferase reporter construct provided by Dimitris Thanos (Biomedical Sciences Research Center, Athens, Greece) was used along with the pRL-TK plasmid (Promega, Madison, Wisconsin, United States) driving constitutive expression of Renilla luciferase. The Dual Luciferase Reporter Assay System from Promega was used for detection of firefly and Renilla luciferase. *Mycobacterium smegmatis* lipomannan, zymosan and Pam_3_CSK_4_ were purchased from InvivoGen (San Diego, California, United States). Sendai virus strain Cantell was grown in 10-day embryonated chicken eggs and titered as previously described [72].

### Cell culture and flow cytometry

DC2.4, MEFs, and HEK293T cells were grown in DMEM supplemented with 10% FBS in a 37°C humidified incubator with an atmosphere of 5% CO_2_. BMDCs were generated by culturing bone marrow cells for 7 days in RPMI supplemented with 10% FBS, 10 ug/mL gentamicin and 25 ng/mL GMCSF, with media changes on days 3 and 5. Non-adherent cells were used for experimentation and were validated to be DCs based on 70% to 80% of the cells staining positive for CD11c and MHCII as measured by flow cytometry. Murine IFNα was purchased from eBioscience (San Diego, California, United States). All flow cytometry staining was performed after a 10-minute incubation in ice cold PBS with Fc receptor block (BD Biosciences, San Jose, California, United States). Antibodies from BD Biosciences directed at the following molecules were used at 1:200 dilution in ice cold PBS for 20 minutes at 4°C: CD11c (#557400), MHCII (#553552), CD80 (#553769), CD86 (#553691) and TLR2 (#562625). All samples were washed three times with ice cold PBS, read on a BD FACSCanto II flow cytometer and analyzed using Flowjo software. For flow cytometry of transfected MEFs, cells were detached from plates by incubating in PBS/10 mM ethylenediaminetetraacetic acid (EDTA) for 20 minutes at 37°C.

### Transfections, metabolic labeling, cell lysis, immunoprecipitations, and Western blotting

HEK293T cells were transfected overnight at 60% confluency using Lipofectamine 2000 (Life Technologies, Carlsbad, California, United States) according to the manufacturer’s instructions. For TLR2 activity experiments, 100 ng of TLR2-expressing plasmids were transfected into wells of a 12-well plate along with 1 ug of NF-κB luciferase reporter plasmid and 50 ng of pRL-TK. For all other experiments, 1 ug or 2 ug of plasmid was transfected for 12-well and 6-well plates, respectively. For cell labeling, alk-16 was added to 37°C DMEM containing 2% charcoal/dextran-filtered fetal bovine serum for a final concentration of 50 uM. For mock-labeled samples, an equivalent volume of DMSO rather than alk-16 solution was added to the labeling media. After replacing the media, labeling was allowed to proceed for one to two hours under standard culture conditions at 37°C or for four hours for proteomic experiments. 2-BP was purchased from Sigma-Aldrich (St. Louis, Missouri, United States), prepared fresh in DMSO for each experiment and used at a concentration of 100 μM. For all alk-16 labeling experiments, cells were washed twice with room temperature PBS and pellets were flash-frozen with liquid nitrogen for storage at −80°C. Cell pellets were lysed with Brij97 buffer (1% Brij 97, 150 mM NaCl, 50 mM triethanolamine, pH 7.4) and cellular debris was cleared by centrifugation for five minutes at 1,000 x g. For immunoprecipitations, anti-FLAG or anti-HA conjugated agarose (Sigma), or anti-GFP antibody (JL-8, Clontech, Mountain View, California, United States) or anti-TLR2 antibody (MAb-mTLR2, InvivoGen, San Diego, California, United States) with protein G agarose (Roche Diagnostics, Indianapolis, Indiana, United States) was used. Western blots for FLAG-tagged proteins were performed with anti-FLAG antibody produced in rabbit (Sigma-Aldrich, St. Louis, Missouri, United States) at a 1:1,000 dilution. Blots for HA-tagged proteins were performed using a polyclonal antibody preparation from Clontech at a 1:1,000 dilution. Blots for fluorescent protein-tagged TLRs were performed with Clontech mouse monoclonal anti-GFP (JL-8) at a 1:2,500 dilution and blots for endogenous mouse TLR2 was performed with anti-TLR2 (MAB1530) from R&D Systems (Minneapolis, Minnesota, United States) at a 1:1,000 dilution.

### Availability of supporting data

MS/MS raw data files have been uploaded to the Peptide Atlas data repository and are freely available for download and analysis. Files for DC2.4 samples can be accessed at PeptideAtlas under dataset identifier PASS00594 [73]. These files correspond to gel slices cut from the first four lanes of Figure 1C. Specifically, files 1 to 7 are from lane 1 (DMSO treated cells), files 8 to 14 are from lane 2 (IFN alpha/DMSO treated cells), files 15 to 21 are from lane 3 (alk-16 treated cells), and files 22 to 28 are from lane 4 (IFN alpha/alk-16 treated cells). Files for MEF samples can be accessed at PeptideAtlas under dataset identifier PASS00595 [74]. These files correspond to gel slices cut from lanes 5 through 8 of Figure 1C. Specifically, files 29 to 35 are from lane 5 (DMSO treated cells), files 36 to 42 are from lane 6 (IFN alpha/DMSO treated cells), files 43 to 49 are from lane 7 (alk-16 treated cells), and files 50 to 56 are from lane 8 (IFN alpha/alk-16 treated cells).

## Additional files

Additional file 1
**Table S1:** Alk-16-labeled proteins identified from DC2.4 cells. **Table S2:** Alk-16-labeled proteins identified from MEFs. **Table S3:** Palmitoylated proteins identified in both DC2.4 cells and MEFs. **Table S4:** Molecular pathway enrichment of palmitoylated proteins found in both MEFs and DC2.4 cells. Additional file 2
**Figure S1:** Confirmation of IRGM1 palmitoylation. **Figure S2:** Ingenuity pathway analysis of palmitoylated proteins identified in both DC2.4 cells and MEFs. **Figure S3:** Confirmation of NEDD4 palmitoylation. **Figure S4:** Identification of CD80 palmitoylation. **Figure S5:** Murine (m) TLR2 is S-palmitoylated on Cys-609. **Figure S6:** Partial conservation of palmitoylation occurring on the human TLRs. **Figure S7:** Expression levels of HA-tagged DHHC constructs. **Figure S8:** TLR2-dependent cytokine responses are decreased by chemical inhibition of palmitoylation in primary human DCs. **Figure S9:** TLR2 S-palmitoylation is required for NF-kB-dependent gene induction.**Figure S10:** S-palmitoylation of TLR2 does not affect its stability.
