# Supplementary material for: Chemoproteomics reveals Toll-like receptor fatty acylation
Source: BMC Biol. 2014 Nov 5;12:91. doi: 10.1186/s12915-014-0091-3 (PMC4240870; doi:10.1186/s12915-014-0091-3)
Supplement: Additional file 2 — Figure S1: Confirmation of IRGM1 palmitoylation. Figure S2: Ingenuity pathway analysis of palmitoylated proteins identified in both DC2.4 cells and MEFs. Figure S3: Confirmation of NEDD4 palmitoylation. Figure S4: Identification of CD80 palmitoylation. Figure S5: Murine (m) TLR2 is S-palmitoylated on Cys-609. Figure S6: Partial conservation of palmitoylation occurring on the human TLRs. Figure S7: Expression levels of HA-tagged DHHC constructs. Figure S8: TLR2-dependent cytokine responses are decreased by chemical inhibition of palmitoylation in primary human DCs. Figure S9: TLR2 S-palmitoylation is required for NF-kB-dependent gene induction.Figure S10: S-palmitoylation of TLR2 does not affect its stability. [file 12915_2014_91_MOESM2_ESM.doc]

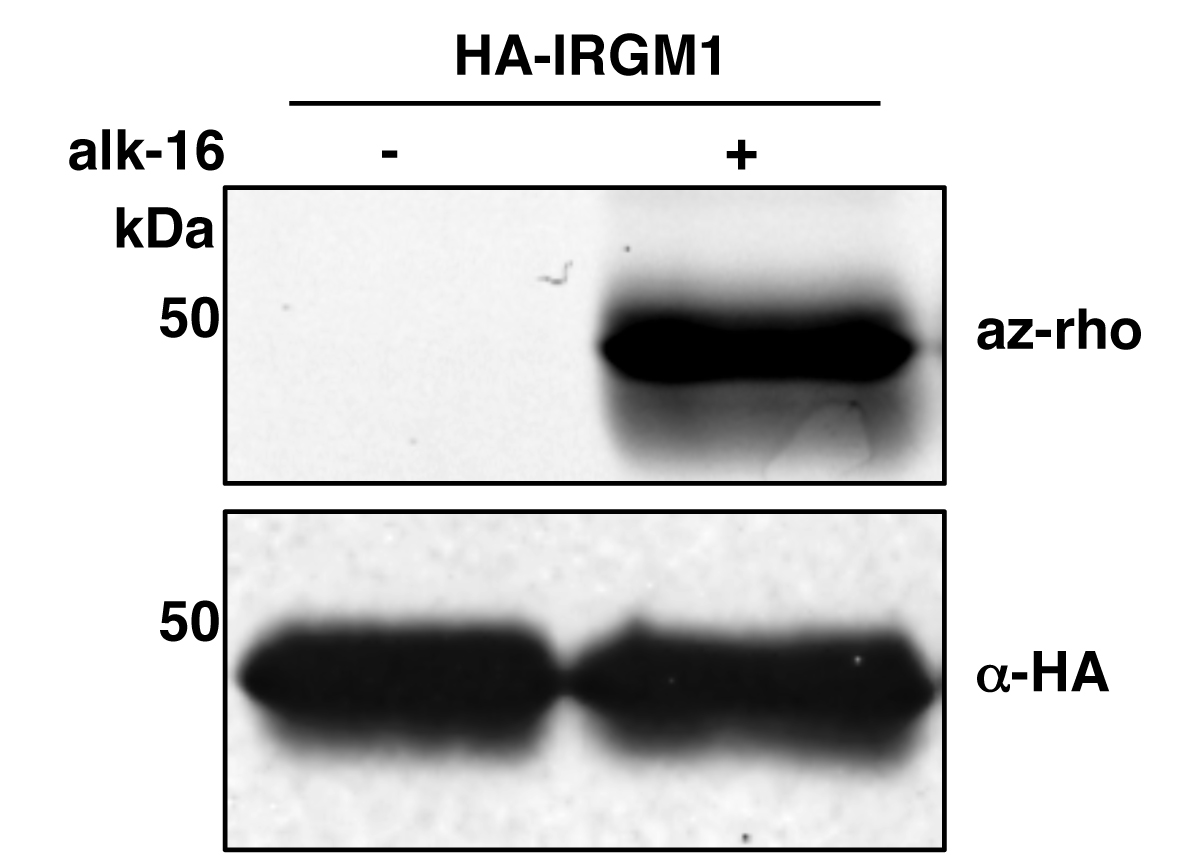


**Figure S1: Confirmation of IRGM1 palmitoylation.** HEK293T cells were transfected with plasmid expressing HA-IRGM1. Cells were labeled with 50 uM alk-16 or DMSO as a control for 1 h. IRGM1 was immunoprecipitated and reacted with az-rho for fluorescent visualization of palmitoylation. Anti-HA Western blotting was performed to compare protein loading.

**
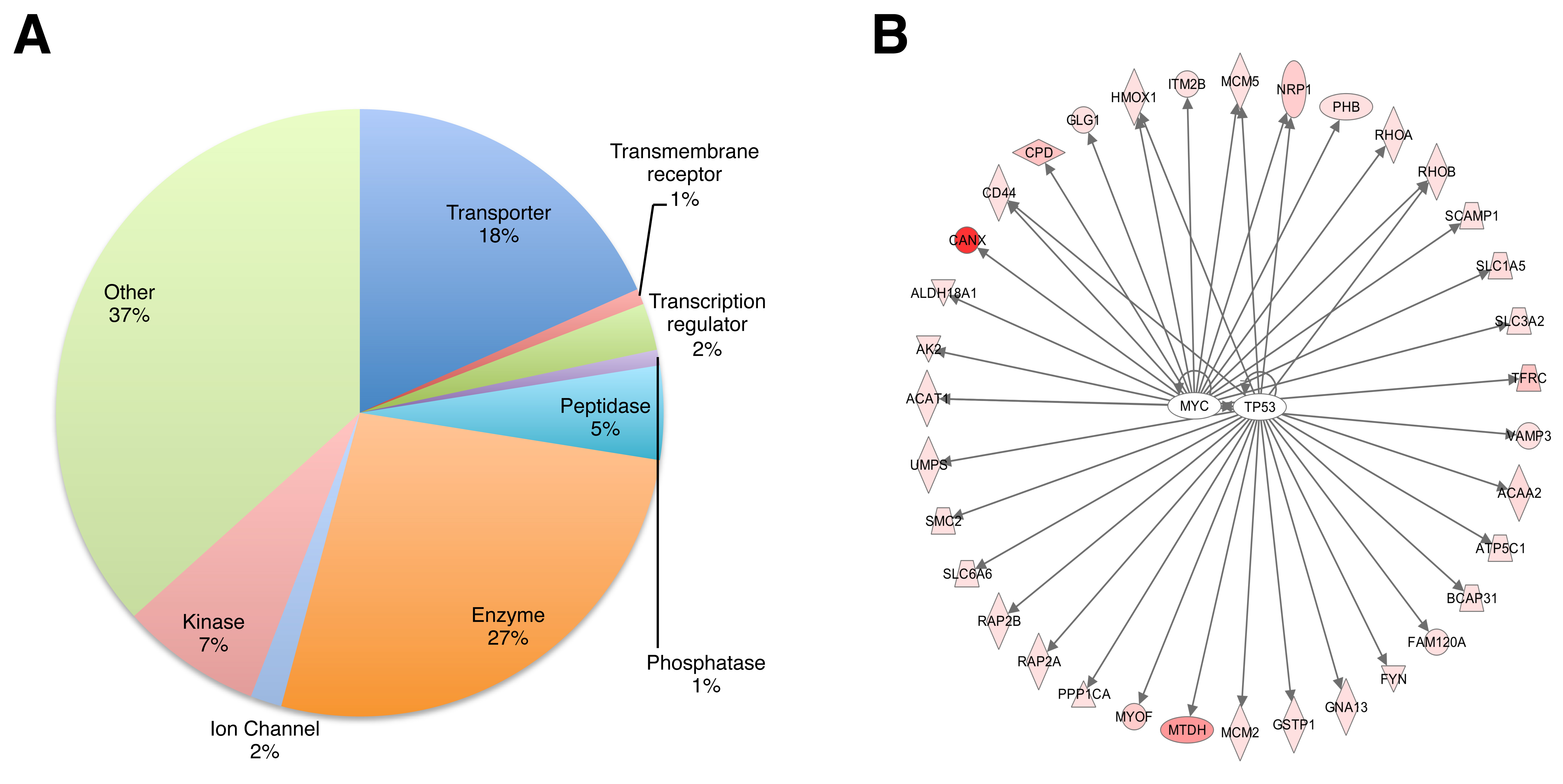
**

**Figure S2: Ingenuity pathway analysis of palmitoylated proteins identified in both DC2.4 cells and MEFs.** A) Function classification of the 121 palmitoylated proteins identified in both cell types. B) Known cancer regulatory proteins, MYC and TP53, are upstream of 29% of the 121 proteins identified in both cell types based on connectivity analysis.


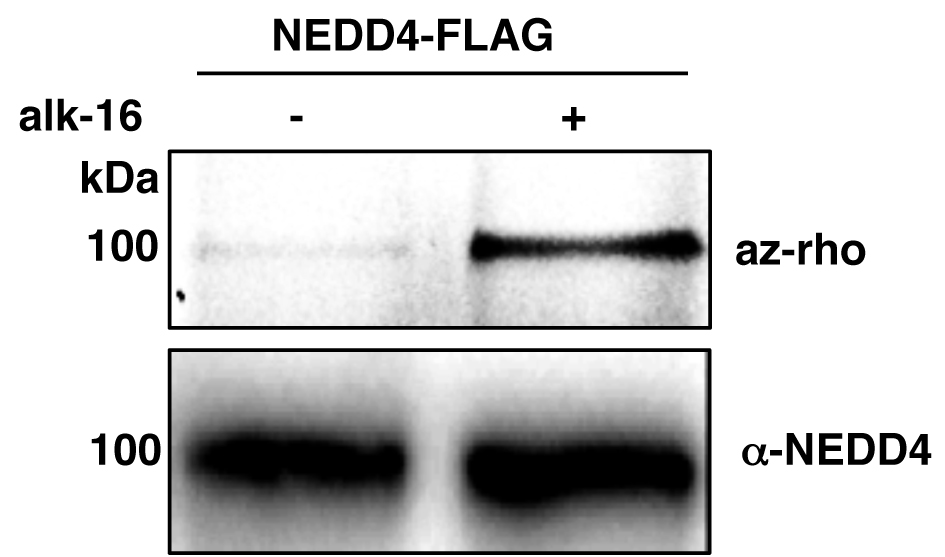


**Figure S3: Confirmation of NEDD4 palmitoylation**. MEFs stably expressing rat NEDD4-FLAG were labeled with 50 uM alk-16 or DMSO as a control for 1 h. Immunoprecipitated NEDD4 was reacted with az-rho for fluorescent visualization of palmitoylation. Anti-NEDD4 Western blotting was performed to confirm comparable protein loading.


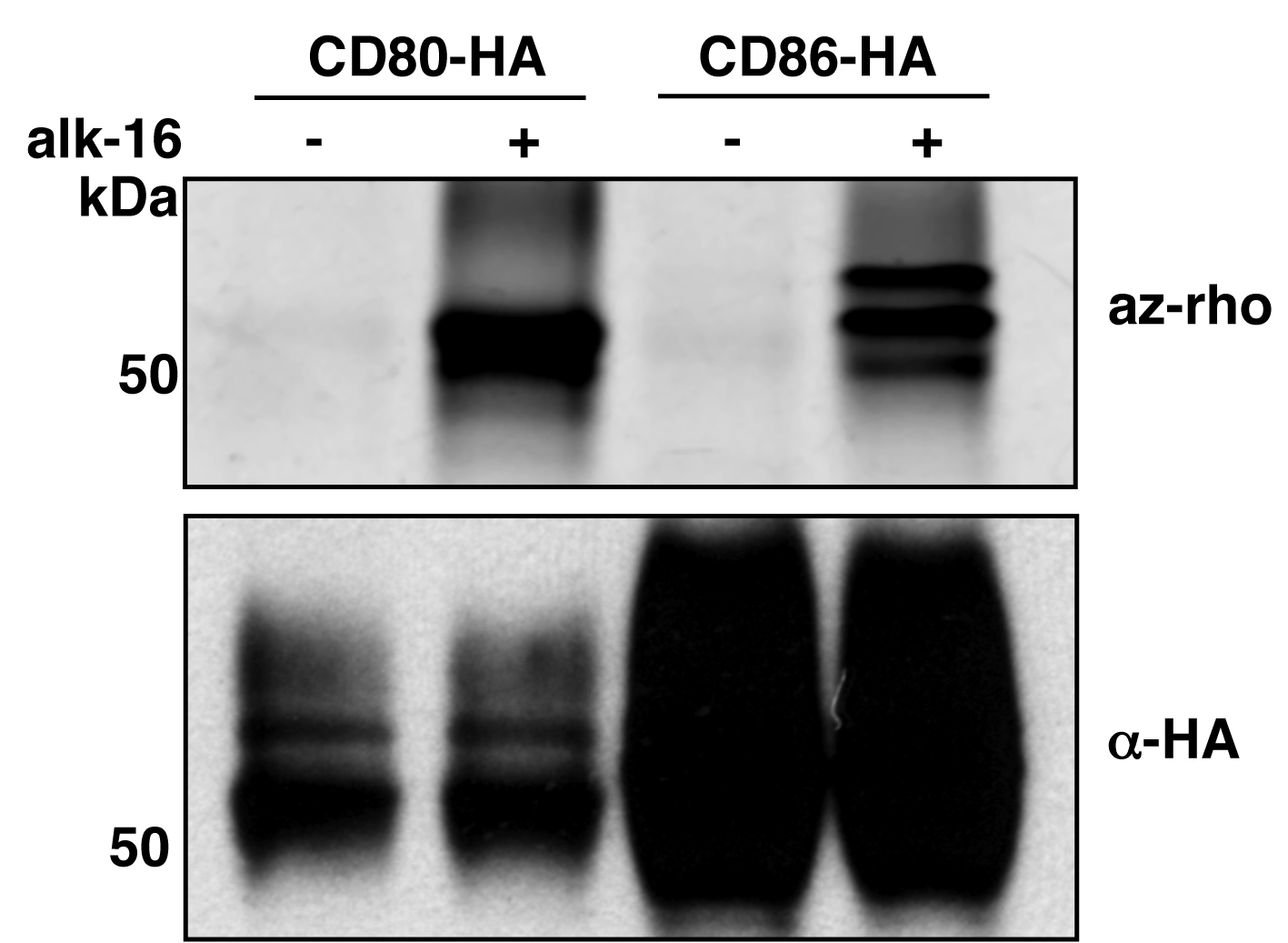


**Figure S4: Identification of CD80 palmitoylation.** HEK293T cells were transfected with plasmids expressing CD80-HA or CD86-HA. Cells were labeld with 50 uM alk-16 or DMSO as a control for 1 h. Immunoprecipitated CD80-HA and CD86-HA were reacted with az-rho for fluorescent visualization of palmitoylation. Anti-HA Western blotting was performed to confirm comparable protein loading.


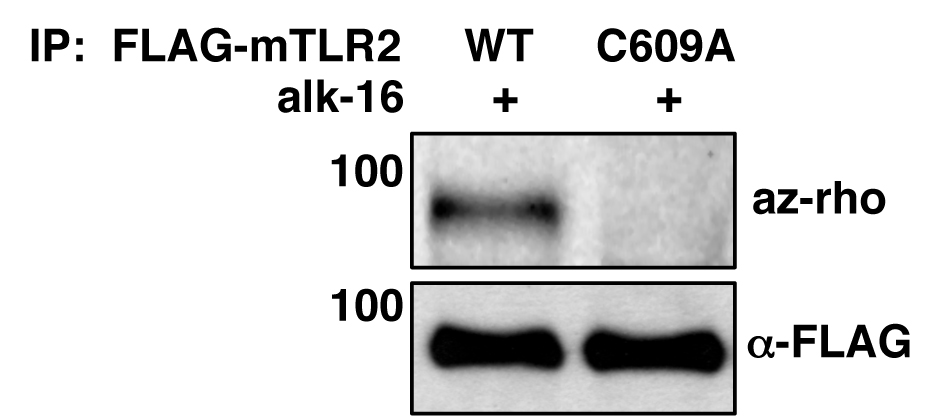


**Figure S5: Murine (m) TLR2 is S-palmitoylated on Cys-609.** HEK293T cells were transfected with FLAG-mTLR2 or FLAG-mTLR2-C609A. Cells were labeled with 50 uM alk-16 for 1 h. TLR2 was immunoprecipitated and reacted with az-rho for fluorescent visualization of palmitoylation. Anti-FLAG Western blotting was performed to confirm comparable protein loading.


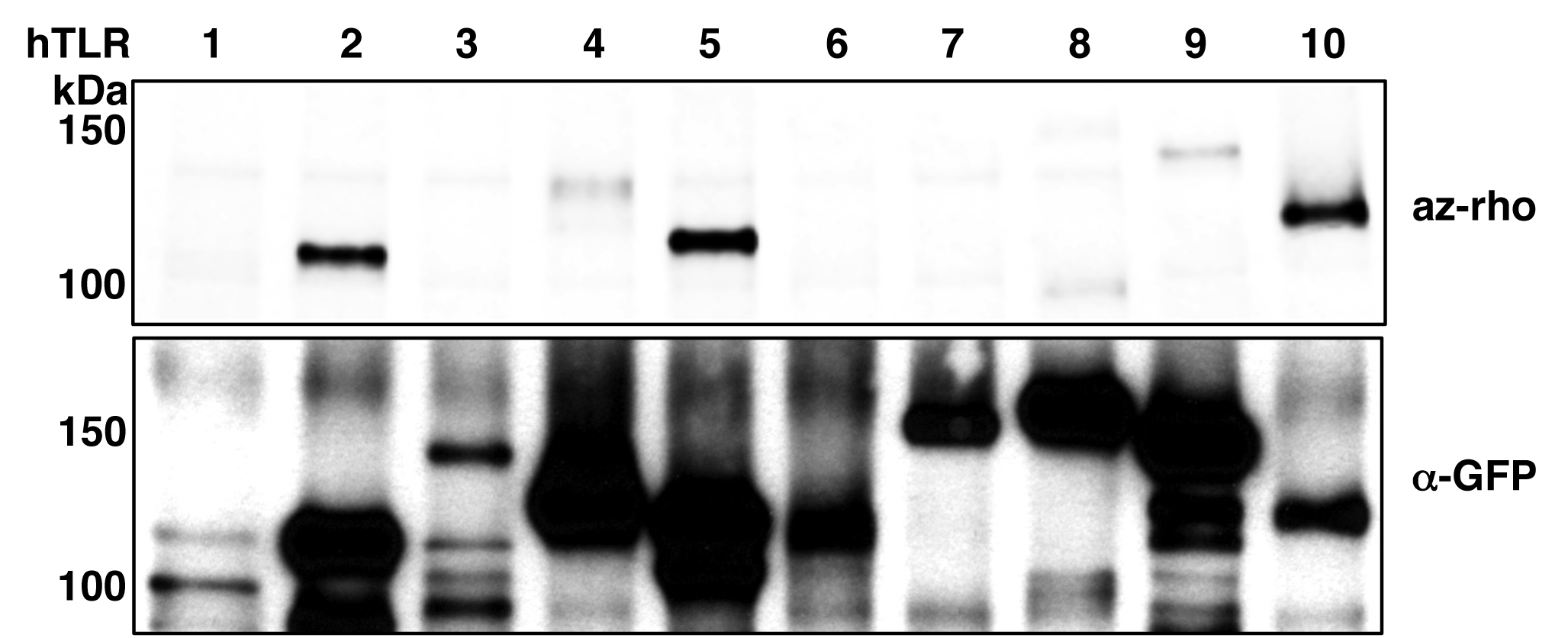


**Figure S6: Partial conservation of palmitoylation occurring on the human TLRs.** Palmitoylation analysis of a panel of human TLRs. HEK293T cells were transfected overnight with plasmids expressing human TLRs 1-10 tagged with fluorescent proteins (YFP or CFP). TLRs were immunoprecipitated and reacted with az-rho for imaging palmitoylation by fluorescent gel scanning. Western blotting with anti-GFP antibodies allowed comparison of protein levels. Results are representative of three identical experiments.


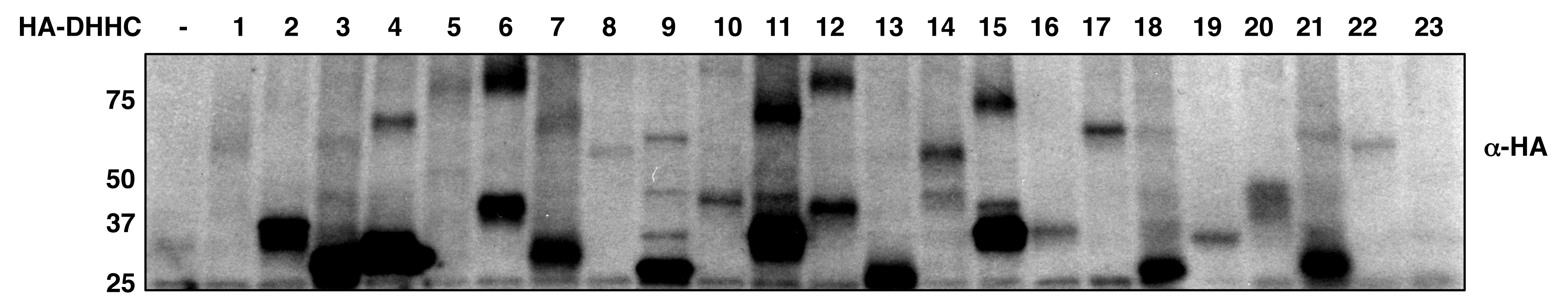


**Figure S7: Expression levels of HA-tagged DHHC constructs.** HA-DHHCs 1-23 or GST were cotransfected into HEK293T cells with FLAG-TLR2. A portion of the cell lysate was subjected to anti-HA immunoporecipitation followed by SDS-PAGE and anti-HA Western blotting. DHHCs are glycosylated proteins appearing as multiple bands. Note that while the expression levels of individual DHHCs vary dramatically, high expression did not directly correlate with activity in Fig 2D,E.


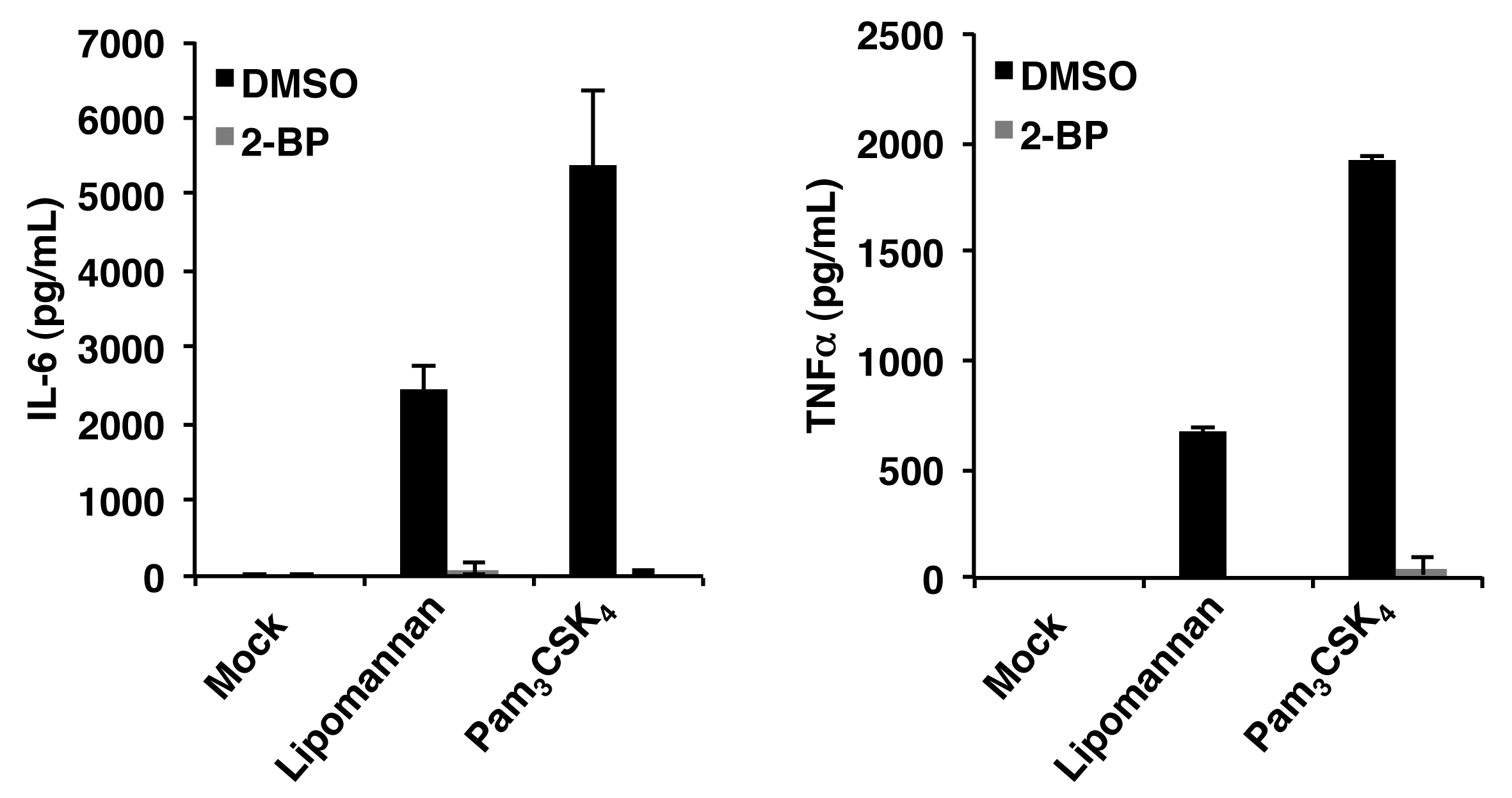


**Figure S8: TLR2-dependent cytokine responses are decreased by chemical inhibition of palmitoylation in primary human DCs.** Human monocyte-derived DCs were treated with 50 uM 2-BP or an equivalent volume of DMSO for 12 h. Cells were then mock treated or treated with lipomannan (4 ug/mL) for an additional 6 h. IL-6 and TNFα levels in cellular supernatants were measured by ELISA. Results are representative of two experiments. Error bars are the standard deviation of triplicate samples.


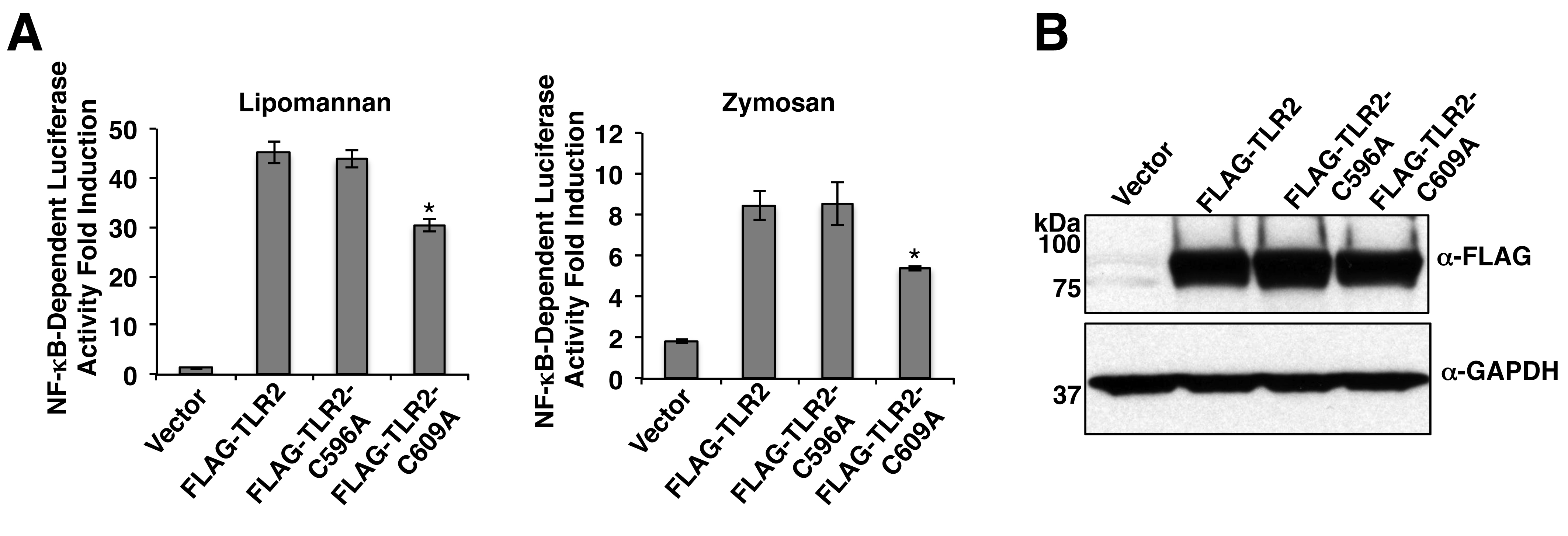


**Figure S9: TLR2 S-palmitoylation is required for NF-kB-dependent gene induction.** A) HEK293T cells were co-transfected with the indicated plasmids along with a reporter construct expressing NF-kB-driven firefly luciferase and a plasmid constitutively expressing renilla luciferase for normalization. Cells were mock treated or treated for 8 h with lipomannan (4 ug/mL) or zymosan (10 ug/mL), and luciferase activity was measured in cell lysates. Results are presented as fold induction over mock treated samples. B) Western blots confirming similar expression of FLAG-TLR2 and mutants in experiments performed in A. Results are representative of more than five experiments. Error bars in A represent standard deviation of triplicate samples. *p<0.001 by Student’s t-test comparing FLAG-TLR2 and FLAG-TLR2-C609A.


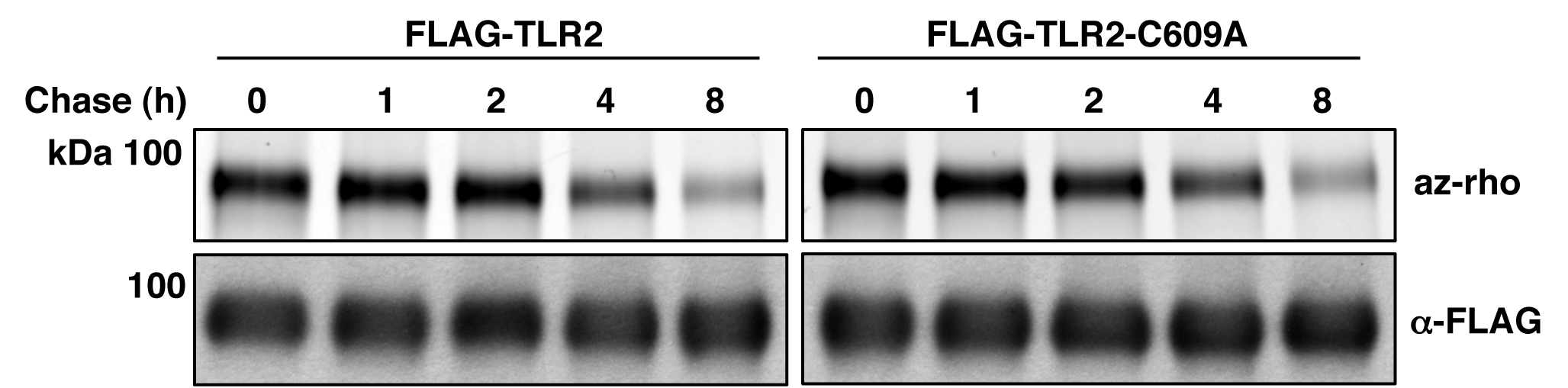


**Figure S10: S-palmitoylation of TLR2 does not affect its stability.** HEK293T cells were transfected with plasmids expressing FLAG-TLR2 or FLAG-TLR2-C609A. Cells were labeled with the alkynyl methionine surrogate homopropargylglycine (HPG) in methionine-free media for 1 h and were chased with methionine-rich media for the indicated times. FLAG-TLR2 was immunoprecipitated and reacted with az-rho for visualization of HPG incorporation and decay of TLR2 over time. Anti-FLAG Western blotting was performed as a loading control. Results are representative of two experiments.
